# Formation and remodeling of immunological niches in solid tumors: organ-specific architectures, inflammatory parallels, and therapeutic reprogramming

**DOI:** 10.3389/fimmu.2026.1903920

**Published:** 2026-07-31

**Authors:** Zhuo Zhang, Yun Dong, Jinchao Li, Youling Shi, Aihua Mei, Miaoxin Fu

**Affiliations:** 1 Sinopharm Dongfeng Stomatological Hospital, Affiliated Stomatological Hospital of Hubei University of Medicine, Shiyan, Hubei, China; 2 Sinopharm Dongfeng General Hospital (Hubei Clinical Research Center of Hypertension), Hubei University of Medicine, Shiyan, Hubei, China

**Keywords:** brain tumors, inflammatory parallels, lung tumors, organ-specific immunity, spatial immunology, therapeutic reprogramming, tumor immunological niche, tumor microenvironment

## Abstract

Tumor immunity is increasingly understood not only through the cellular composition of the tumor microenvironment, but also through spatially organized local niches that can help determine where immune recognition is initiated, constrained, suppressed, or therapeutically restored. This review develops an organ-conditioned and evidence-graded framework for tumor immunological niches as spatially localized, interaction-dependent, functionally consequential, and dynamically remodeled units. Rather than reproducing a general taxonomy of cancer immune niches, we focus on how shared niche-forming mechanisms are implemented differently by organ-specific tissue rules. We first distinguish the niche concept from broad tumor microenvironment descriptions, immune infiltration, immune compartments, and tertiary lymphoid structures. We then propose an operational evidence hierarchy that separates spatial association, recurrent interaction, functional consequence, perturbation-based validation, and therapeutic actionability. Next, we synthesize core mechanisms of niche formation, including tumor-intrinsic signaling, stromal and extracellular-matrix scaffolding, chemokine and cytokine wiring, vascular-hypoxic-metabolic boundaries, myeloid-centered suppression, and tissue-resident immune imprinting. Brain and lung tumors are treated as two organizing paradigms: immune-restricted niche architecture in the central nervous system and inflammation-primed niche architecture in the lung. Oral, liver, and pancreatic tumors are discussed comparatively to show how mucosal-microbial, tolerogenic-metabolic, and desmoplastic immune-exclusion rules reshape shared niche mechanisms. We further separate tumor-specific evidence, inflammatory analogies, and hypothesis-generating parallels to avoid overextending chronic inflammation or fibrosis models. Finally, we examine how niche architecture produces T-cell exclusion, antigen-presentation failure, suppressive myeloid-stromal feedback, and therapeutic resistance, and how release, access, and licensing strategies may guide organ-tailored combinations when supported by adequate evidence. This review argues that tumor immunological niches may serve as meso-level analytical units linking spatial organization, organ context, evidence strength, inflammatory parallels, and therapeutic vulnerability when their evidentiary status is explicitly defined.

## 1 Introduction: from the TME to organ-conditioned immunological niches

The tumor microenvironment (TME) concept has been indispensable for moving tumor immunology beyond a tumor-cell-autonomous view of cancer. It established that malignant progression, metastasis, and treatment response depend on reciprocal interactions among cancer cells, immune populations, stromal cells, vascular networks, soluble mediators, and metabolic circuits (1–3). However, as single-cell, spatial transcriptomic, spatial proteomic, and multiplex imaging approaches have increased the resolution of tumor analysis, an important limitation of the broad TME framework has become more visible. The overall abundance of immune cells, bulk inflammatory signatures, or general stromal classification cannot fully explain why sharply adjacent regions in the same tumor may differ in immune activation, immune exclusion, antigen-presentation capacity, myeloid suppression, or therapeutic response (3–6).

The concept of the tumor immunological niche addresses this analytical gap. It should not be treated as a simple synonym for the TME. Rather, it provides an intermediate level of analysis between global microenvironmental description and single-cell phenotyping. In this review, a tumor immunological niche refers to a spatially organized, interaction-dependent, and functionally consequential local unit formed by coordinated relationships among tumor cells, immune cells, stromal and vascular elements, extracellular matrix, soluble signals, metabolic conditions, and organ-specific tissue programs (3, 7). A niche may support antitumor immunity, as in local structures that sustain antigen presentation, T-cell activation, or tertiary lymphoid organization (8–14). It may also stabilize immune exclusion, suppressive myeloid programming, stemness, metastatic competence, or therapy resistance (15–17).

This shift matters because immune function in solid tumors is rarely homogeneous. T cells do not become effective merely by entering tumor tissue; their function depends on whether they are positioned near antigen-presenting or stimulatory cells, protected from suppressive ligands and metabolites, able to cross stromal and vascular barriers, and maintained long enough to exert effector activity. Chemokines, cytokines, stromal scaffolds, vascular access points, extracellular matrix architecture, oxygen gradients, and nutrient availability therefore do not merely accompany immune infiltration. They organize the local sites where immune responses are permitted, distorted, or blocked (3, 18–23).

An organ-specific perspective is essential to this niche framework. Shared mechanisms such as myeloid suppression, stromal remodeling, chemokine-guided positioning, vascular dysfunction, and metabolic constraint are not implemented identically across organs. They are filtered through tissue architecture, resident immune programs, barrier function, neural input, microbial exposure, chronic inflammation, and regenerative history. Brain tumors illustrate an immune-restricted architecture shaped by central nervous system barriers, resident microglia, perivascular interfaces, glial-immune crosstalk, hypoxic and necrotic microdomains, and limited support for effective T-cell activation (24–26). Lung tumors illustrate an inflammation-primed architecture, where continuous environmental exposure, epithelial injury-repair cycles, chronic pulmonary disease, lymphoid structures, and myeloid-stromal remodeling may precondition tumor-associated immune niches (27–36).

This review therefore narrows a broad field into a focused argument: tumor immunological niches are organ-conditioned, spatially organized, and potentially therapeutically remodelable analytical units whose functional and clinical significance should be judged according to evidence strength. The following sections first define the conceptual boundaries of the niche perspective, then synthesize the core mechanisms of niche formation and stabilization, then examine brain and lung tumors as two paradigmatic organ contexts. Oral, liver, and pancreatic tumors are then used as comparative extensions, not as separate exhaustive reviews. The final section integrates functional consequences and therapeutic reprogramming, emphasizing that effective niche-guided therapeutic interpretation requires identifying the dominant local barrier, assessing the strength of causal evidence, and selecting organ-tailored combinations rather than simply intensifying checkpoint blockade.

### 1.1 Positioning of this review in relation to recent immune niche frameworks

A recently published review by Slingerland, Runderkamp, and Thommen provided a broad and mechanistically organized taxonomy of immune niches in cancer, including immune triads, stem lymphoid-myeloid niches, nonstem lymphoid-myeloid niches, and tertiary lymphoid structures, and connected these spatial architectures to antitumor immunity, prognosis, and immunotherapy response (37). The present review does not aim to reproduce that taxonomy. Instead, its specific contribution is to ask how organ-specific tissue rules condition the formation, functional output, evidence strength, and therapeutic actionability of tumor immunological niches. We therefore organize the manuscript around three focused contributions: first, a practical evidence hierarchy for distinguishing spatial immune patterns from functionally validated niches; second, a side-by-side comparison of brain and lung tumors as immune-restricted and inflammation-primed paradigms; and third, an evidence-qualified discussion of inflammatory parallels and therapeutic reprogramming. This positioning also guides the selection of examples: primary spatial or mechanistic studies are emphasized where they clarify organ-conditioned niche logic and where the level of evidence supporting each niche claim can be stated explicitly rather than simply adding another catalogue of immune components.

**Figure 1:**
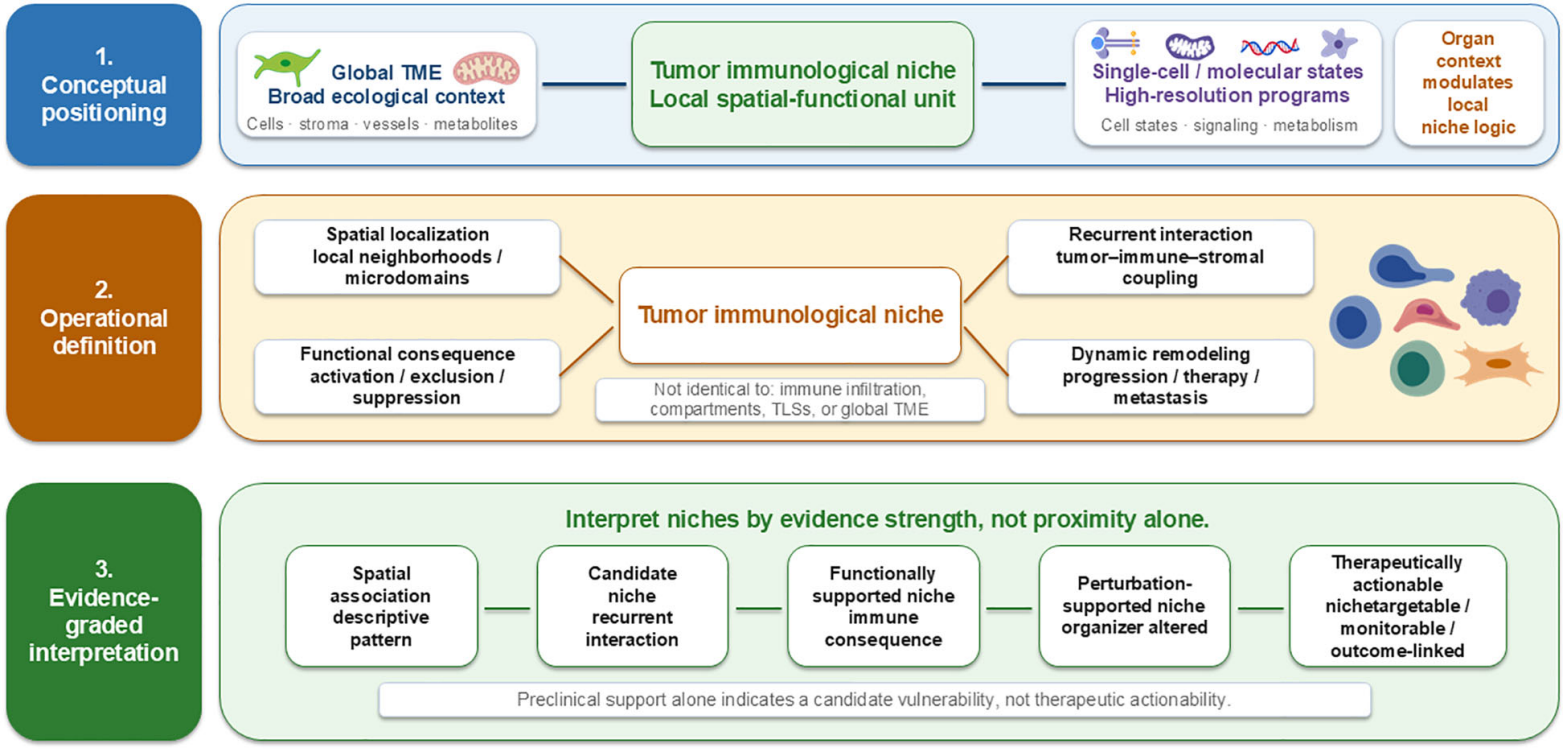
Conceptual framework of organ-conditioned tumor immunological niches. The schematic positions tumor immunological niches as meso-level spatial-functional units between the global tumor microenvironment and high-resolution single-cell or molecular states. It summarizes the operational definition of tumor immunological niches and emphasizes evidence-graded interpretation, distinguishing spatial association, candidate niches, functionally supported niches, perturbation-supported niches, and therapeutically actionable niches. Detailed niche-forming mechanisms, organ-specific examples, functional consequences, and therapeutic strategies are summarized in the text, **Box 1**, and **Tables 1**–**5**. This image is an author-prepared conceptual schematic, not an AI-generated image, and does not represent quantitative spatial data. TME, tumor microenvironment; TLS, tertiary lymphoid structure.

**Figure 1** summarizes the conceptual logic of this review by emphasizing three distinctions: what is insufficient on its own to define a niche, what minimally defines a tumor immunological niche, and how evidence strength should be graded before therapeutic implications are inferred. It should therefore be read as an author-prepared conceptual schematic rather than as a quantitative spatial map.

Box 1 **Operational evidence framework for identifying tumor immunological niches**

**Table:** 

Evidence level	Minimum evidence	Supported interpretation	Typical methods	Interpretive caution
Spatial association	Repeated local enrichment or regional positioning of tumor, immune, stromal, vascular, or metabolic features.	Spatial immune pattern, compartment, or neighborhood.	Spatial transcriptomics, multiplex imaging, pathology-based neighborhood mapping.	Does not prove interaction or causality.
Recurrent interaction	Reproducible contact, proximity, ligand-receptor wiring, or neighborhood structure among niche components.	Interaction-dependent candidate niche.	Cellular neighborhood analysis, spatial proteomics, single-cell/spatial integration.	Still inferential unless linked to function.
Functional consequence	Local arrangement correlates with activation, exclusion, suppression, immune ignorance, progression, prognosis, or response.	Functionally consequential niche.	Clinical association, immune-function readouts, longitudinal sampling.	Association should not be overstated as causality.
Perturbation validation	Disrupting a niche organizer changes the predicted immune output.	Mechanistically supported niche.	Animal models, organoids, co-culture, genetic or pharmacologic perturbation.	Model context and organ specificity must be stated.
Therapeutic actionability	Niche state is targetable, monitorable, and linked to treatment outcome or pharmacodynamic change.	Actionable therapeutic niche.	Clinical biomarker studies, trials, spatial pharmacodynamics.	Use only when evidence exceeds conceptual rationale.

**Table 1:** Core mechanisms of tumor immunological niche formation and stabilization.

Mechanism	Niche-level function	Interpretive emphasis	Representative references	Evidence type/status
Tumor-intrinsic programs	Externalize malignant-cell states into local tissue through oncogenic, epigenetic, secretome, stress-associated, and metabolic outputs.	Use selected driver examples only when they explain niche assembly rather than tumor growth alone.	(1, 44–46, 53–56, 76)	Review-level synthesis plus selected tumor-intrinsic mechanistic evidence; not sufficient alone for niche causality.
Stroma and ECM	Create immune-permissive, immune-excluded, or transitional compartments through CAF states, matrix topology, stiffness, and collagen organization.	Avoid repeating CAF evidence across every cancer type; retain spatially informative examples.	(23, 48, 52, 58, 59, 61–63, 128, 137–139)	Primary spatial and mechanistic evidence when CAF/ECM architecture is linked to T-cell exclusion, stromal barriers, or treatment response.
Chemokine/cytokine gradients	Wire recruitment, retention, proximity, and immune-cell interaction.	Interpret chemokines and cytokines as spatial organizing cues rather than generic soluble factors.	(18, 41, 51, 64–68)	Mechanistic and review-level evidence; spatial relevance depends on local gradients, cellular neighborhoods, and functional readouts.
Vascular-hypoxic-metabolic boundaries	Regulate leukocyte entry, oxygenation, acidity, nutrient access, drug penetration, and effector-cell persistence.	Link vascular dysfunction, hypoxia, and metabolism to spatial immune function, not merely to stress biology.	(17, 43, 69–77, 90, 98, 132, 153)	Primary spatial, preclinical mechanistic, and biomarker evidence depending on cancer type and treatment context.
Myeloid-centered suppression	Connect TAMs, MDSCs, neutrophils, suppressive lymphocytes, exhausted T cells, and stromal circuits into suppressive niches.	Move beyond M1/M2; emphasize lineage, localization, plasticity, metabolic state, and intervention points.	(2, 78–82, 89, 111, 134, 151)	Primary spatial and mechanistic evidence where TAM/MDSC/neutrophil positioning is linked to immune suppression or resistance.
Tissue-resident imprinting	Translates shared mechanisms into organ-specific immune architectures through local resident cells and exposure histories.	Use as the bridge to brain and lung paradigms.	(25, 83–89)	Primary spatial and review-level evidence; organ imprinting is strongest when linked to resident immune programs and tissue-specific exposure histories.

**Table 2:** Organ-specific niche architectures across selected solid tumors.

Tumor context	Dominant niche logic	Tissue rules and organizers	Main immune consequence	Representative references	Evidence type/status
Brain tumors	Immune-restricted architecture	CNS barriers; microglia and border macrophages; perivascular, hypoxic, and necrotic microdomains; neuron-glial-immune coupling	Myeloid dominance; low T-cell support; impaired antigen presentation; limited checkpoint efficacy	(24–26, 38, 44, 90–106)	Primary spatial evidence and preclinical therapeutic rationale; clinical actionability remains limited in many brain tumors.
Lung tumors	Inflammation-primed architecture	Inhaled exposure; epithelial injury-repair cycles; AT2 programs; chronic pulmonary disease; TLS and immune compartmentalization	Mixed immune activation and suppression; TLS maturation dependence; resistance through stromal-myeloid and antigen-presentation programs	(27–36, 63, 84, 87, 88, 107–112)	Primary spatial evidence plus clinical biomarker evidence in selected immunotherapy contexts; heterogeneous across cohorts.
Oral/HNSCC	Mucosal-microbial barrier niche	Barrier disruption; dysbiosis; epithelial-myeloid crosstalk; CAF and macrophage neighborhoods	Spatially restricted T-cell dysfunction and microbial-inflammatory immune modulation	(5, 113–116, 125–127, 154)	Primary spatial evidence and clinical biomarker evidence, with microbial-inflammatory interpretation partly hypothesis-generating.
Liver tumors	Tolerogenic-metabolic niche	Portal exposure; viral persistence; fibrosis; hepatic stellate cells; endothelial and macrophage programs; bile acid and metabolic stress	Tolerance-associated suppression, fibrosis-linked immune exclusion, and metabolic resistance	(12, 61, 62, 70, 117–120, 128–130, 139, 155)	Single-cell/spatial evidence plus clinical biomarker and preclinical rationale; organ tolerance and metabolism require functional validation.
Pancreatic tumors	Desmoplastic immune-excluded niche	Dense ECM; CAF heterogeneity; hypoxia; macrophage and neutrophil circuits; metabolic stress; delivery barriers	T-cell exclusion, stromal drug barrier, and poor checkpoint responsiveness	(15, 16, 66, 76, 121–124, 131, 132)	Mechanistic and preclinical therapeutic rationale with spatial support; clinical actionability remains context-dependent.

**Table 3:** Functional consequences and therapeutic vulnerabilities.

Niche outcome	Functional meaning	Representative organizers	Therapeutic implication	Representative references	Evidence type/status
Spatial immune exclusion	T cells are present but retained away from tumor-cell contact zones.	CAF/ECM remodeling, myeloid-neutrophil circuits, vascular barriers, hypoxic-metabolic stress	Stromal remodeling, ECM targeting, myeloid modulation, vascular normalization	(23, 44, 59–63, 111, 137–139)	Primary spatial evidence and clinical biomarker evidence when exclusion correlates with prognosis or treatment response.
Immune ignorance	Antigens are present but fail to trigger productive recognition.	MHC repression, dendritic-cell dysfunction, impaired IFN signaling, altered innate immune sensing	Restore antigen presentation, DC licensing, and IFN/MHC pathway integrity	(12, 105, 112, 141–143)	Mechanistic and clinical biomarker evidence when MHC/IFN/APC defects are linked to immune escape or resistance.
Myeloid-stromal suppression	Fibroblast and myeloid cells form reciprocal suppressive circuits.	CAF-TAM/MDSC/neutrophil loops, TGF-β, CXCL12, inflammatory signals, and metabolic signals	Break feedback loops; reprogram TAMs/CAFs; combine with checkpoint blockade	(2, 23, 61–63, 66–68, 78–81, 111, 137–140, 145)	Primary spatial and mechanistic perturbation evidence when fibroblast-myeloid circuits are causally tested.
Therapy-induced resistance	Treatment selects or induces suppressive spatial states.	Antigen loss, hypoxia, stromal compensation, resistant tumor states, suppressive inflammation	Longitudinal spatial monitoring and adaptive combinations	(33, 57, 77, 101–103, 110, 119, 123, 124, 139, 146–150)	Clinical biomarker and preclinical therapeutic evidence; causal attribution should be made cautiously.
Therapeutic niche remodeling	Treatment opens immune-permissive windows.	TLS maturation, myeloid activation, vascular normalization, metabolic relief, antigen-presentation recovery	Organ-tailored combinations guided by spatial biomarkers	(6, 12, 14, 15, 76, 78, 98, 104, 105, 112, 135, 141, 142, 149–153)	Preclinical therapeutic rationale or early clinical evidence depending on strategy; requires spatial pharmacodynamic validation.

**Table 4:** Side-by-side comparison of brain and lung tumor immunological niche architectures.

Dimension	Brain tumors	Lung tumors
Dominant tissue rule	CNS immune restriction, specialized barriers, and resident myeloid geography.	Environmental exposure, epithelial injury-repair, and chronic inflammatory conditioning.
Dominant resident immune cells	Microglia, CNS-border macrophages, monocyte-derived macrophages, and perivascular myeloid populations.	Alveolar macrophages, airway/alveolar immune populations, lymphoid aggregates, and recruited myeloid cells.
Barrier structures	Blood-brain barrier, perivascular interfaces, CNS borders, hypoxic and necrotic microdomains.	Airway and alveolar barriers, vascular-inflammatory interfaces, TLSs, and macrophage-CAF neighborhoods.
Common suppressive circuits	Myeloid dominance, impaired antigen presentation, metabolic stress, limited T-cell support, and hypoxia/necrosis-associated suppression.	CAF-myeloid barriers, chronic inflammatory suppression, antigen-presentation heterogeneity, exhausted T cells, and therapy-induced remodeling.
Spatial technologies emphasized	Spatial immune mapping, single-cell/spatial profiling of brain tumors, and multiomic studies of CNS immune borders.	Spatial multi-omics, single-cell mapping across premalignant-to-invasive lung cancer, TLS maturation analysis, and treatment-response spatial profiling.
Therapeutic vulnerabilities	Myeloid reprogramming, antigen-presentation restoration, STING/type I IFN activation, vascular access, and neuron-glial-immune circuit disruption.	Checkpoint-responsive subsets, TLS maturation support, CAF-myeloid remodeling, antigen-presentation correction, and metabolic or vascular modulation.
Evidence maturity	Strong spatial and preclinical rationale; limited clinical success for checkpoint blockade alone in many glioblastoma settings.	More clinical biomarker and immunotherapy-response evidence in selected NSCLC contexts, but substantial heterogeneity remains.
Unresolved questions	How can adaptive immunity be sustained in CNS-restricted and myeloid-dominated niches?	How can productive TLSs and immune-permissive neighborhoods be distinguished from nonfunctional lymphoid aggregates or suppressive inflammation?

**Table 5:** Therapeutic strategies for tumor immunological niche remodeling: evidence status and organ-specific considerations.

Therapeutic logic	Strategy/example classes	Main niche barrier targeted	Evidence status	Organ-specific considerations	Representative references
Release	PD-1/PD-L1 or broader checkpoint blockade.	Inhibitory immune signaling and exhausted T-cell states	Established clinical evidence in selected tumors; response remains niche- and organ-dependent	Stronger clinical relevance in subsets of lung tumors than in glioblastoma; requires antigen presentation and immune access.	(57, 109, 110, 120, 143)
Release	Myeloid reprogramming and macrophage/MDSC-targeted strategies.	TAM/MDSC-centered suppression and myeloid-stromal feedback	Mostly preclinical or early clinical depending on agent and tumor type	Particularly relevant to brain and pancreatic tumors, where myeloid dominance is a major barrier.	(78, 82, 102–104, 151)
Access	CAF/TGF-β modulation, ECM remodeling, and stromal state switching.	Physical exclusion, fibroblast-myeloid barriers, collagen and matrix constraints	Mixed clinical/preclinical evidence; effects may be context-dependent	Important in lung, oral/HNSCC, liver fibrosis-associated tumors, and PDAC desmoplasia.	(23, 59–63, 121, 128, 131, 137, 139, 152)
Access	Vascular normalization, hypoxia correction, and local delivery strategies.	Leukocyte entry, drug delivery, hypoxic suppression, and perivascular immune restriction	Clinical or preclinical depending on strategy and tumor context	Especially relevant where perivascular or hypoxic niches shape immune exclusion, including brain and liver tumors.	(17, 69–73, 90, 98, 132)
Licensing	STING/type I IFN activation, dendritic-cell activation, and IFN/MHC recovery.	Defective antigen recognition and immune ignorance	Preclinical, early clinical, or biomarker-supported evidence depending on context	Promising for brain tumors and antigen-presentation-defective lung tumors, but clinical maturity remains uneven.	(12, 105, 141–143)
Licensing	TLS maturation and lymphoid-structure support.	Local adaptive immune coordination and antigen presentation	Clinical biomarker and preclinical rationale; therapeutic induction remains developing	Most relevant where TLS maturation predicts immune responsiveness, especially lung cancer and HNSCC.	(8–14, 31, 32, 112, 135)
Cross-cutting	Metabolic modulation involving lactate, HIF, lipid, or nutrient pathways.	Metabolic immune suppression and effector-cell dysfunction	Mostly preclinical or early clinical; biomarker selection is needed	Relevant in hypoxic brain niches, liver metabolic niches, and desmoplastic or metabolically constrained PDAC.	(2, 46, 71–77, 119, 123, 130, 153)

## 2 Conceptual framework: what defines a tumor immunological niche?

### 2.1 A meso-level spatial-functional unit

A tumor immunological niche should be defined precisely enough to avoid conceptual inflation. It is related to the TME, immune infiltration, immune compartments, and tertiary lymphoid structures (TLSs), but it is identical to none of them. Four features are central. First, a niche is spatially localized. Spatial analyses of primary and metastatic tumors show that immune lineages, activation states, and cellular interactions are not evenly distributed, but are instead organized into local neighborhoods and microdomains that can correlate with prognosis, immune escape, or therapeutic response (38–40). Spatial localization is therefore not an illustrative layer added after conventional TME analysis; it is part of the definition of the niche itself.

Second, a niche is interaction-driven. It is not defined by one cell type alone, but by the sustained coupling among tumor cells, immune cells, stromal cells, vascular elements, soluble mediators, extracellular matrix, metabolites, and, in some tissues, microbial or neural cues (3, 18, 41, 42). A macrophage-rich area, a fibroblast-rich interface, or a hypoxic region becomes a niche only when the local interaction pattern produces a reproducible functional immune consequence. This requirement distinguishes a niche from a simple colocalization map.

Third, a niche is functionally specific. An immune-rich region is not automatically an antitumor niche. Depending on the local interaction pattern, the same immune or stromal component may support cytotoxicity, myeloid suppression, immune ignorance, metastatic colonization, or treatment resistance (16, 17, 23, 43, 44). Fourth, a niche is dynamically plastic. It is remodeled during tumor initiation, progression, therapeutic pressure, and metastatic spread. Niche plasticity includes local transitions in stromal architecture, myeloid polarization, dendritic-cell function, T-cell exhaustion, vascular permeability, metabolic restriction, and distant-organ preconditioning (45–48).

### 2.2 Boundaries from neighboring concepts

These criteria clarify what immunological niches are not. Immune infiltration describes whether immune cells are present and abundant, but it does not explain whether they form functional local relationships. Immune compartments describe anatomical or histological regions such as tumor nests, stroma, invasive fronts, hypoxic cores, and perivascular zones, but they become niches only when their interaction logic and functional output are specified (17, 43, 49). TLSs are important lymphoid niches, especially when mature germinal-center-like structures support local antigen presentation and adaptive immunity, but they are only one specialized subtype. Many suppressive myeloid, hypoxic, perivascular, microbial, or fibroblast-rich niches lack TLS architecture yet still organize immune function (8–14).

The value of this conceptual boundary is methodological as well as biological. If immune niches are defined by local interactions and functional consequences, then static cell abundance is insufficient evidence. Adequate niche analysis must connect cellular state, spatial position, interaction partners, tissue constraints, and functional readouts. Spatial transcriptomics, multiplex imaging, single-cell multi-omics, computational neighborhood analysis, organoid and co-culture systems, and spatially informed animal models therefore become complementary tools rather than interchangeable methods (4, 19–22, 40, 50).

This definition is intended to prevent the niche concept from expanding into a catalogue of unrelated immune mechanisms. A tumor-cell signal, macrophage state, fibroblast subset, chemokine gradient, vascular interface, or metabolic constraint becomes niche-forming only when it participates in a local organization that produces a durable immune consequence. The central analytical question is therefore not simply “which cells or pathways are present?” but “how are they locally arranged, maintained, and associated with immune activation, exclusion, suppression, immune ignorance, or therapeutic vulnerability?”

### 2.3 Evidence hierarchy for niche identification

Because the niche concept depends on spatial organization and functional consequence, evidence for a tumor immunological niche should not stop at cell abundance or spatial proximity (3, 19). In this review, we use a five-level evidence hierarchy to distinguish descriptive spatial patterns from functionally or therapeutically validated niches. The first level is spatial association, showing that specific tumor, immune, stromal, vascular, or metabolic features repeatedly occupy defined local regions (3, 19). The second level is recurrent cellular interaction, showing that these components form reproducible neighborhoods or contact patterns rather than random colocalization (21, 22). The third level is functional consequence, linking the local arrangement to immune activation, exclusion, suppression, immune ignorance, metastatic progression, or therapeutic response (22, 23, 44). The fourth level is perturbation-based validation, demonstrating that disruption of a niche organizer alters the predicted immune outcome (44). The fifth level is therapeutic actionability, showing that the niche state is targetable, monitorable, and linked to treatment outcome or pharmacodynamic change in a clinically meaningful way (3, 23, 44). This hierarchy is important because spatial maps, although powerful, do not by themselves prove causal niche function or therapeutic actionability.

Operationally, this review reserves the term tumor immunological niche for spatially localized, recurrent interactions linked to an evidence-supported immune consequence. Spatial proximity alone is described as a spatial immune pattern, compartment, or neighborhood, whereas recurrent interactions with only inferred functional consequences are described as candidate niches. Claims of therapeutic actionability are reserved for niche states that are targetable, monitorable, and linked to treatment outcome or pharmacodynamic change. Strategies supported only by preclinical rationale are described as candidate therapeutic vulnerabilities or preclinical therapeutic rationales rather than clinically actionable niches. This operational evidence framework is summarized in **Box 1**.

## 3 Core mechanisms of niche formation and stabilization

Tumor immunological niches are best interpreted as emerging through coupled and recursive processes rather than through the accumulation of isolated mechanisms. Tumor-intrinsic programs can provide initiating signals; stromal and extracellular matrix (ECM) remodeling can create structural and biochemical scaffolds; chemokine and cytokine gradients can recruit and position immune populations; vascular dysfunction, hypoxia, and metabolic constraints can define local functional boundaries; myeloid-centered programs may stabilize suppressive feedback; and tissue-resident immune programs may imprint organ specificity (51–53). These mechanisms are recurrent across solid tumors, but their relative importance varies by organ context. Niche formation should therefore be interpreted not as the sum of isolated pathways, but as the emergence of spatially organized and potentially functionally consequential immune ecology.

### 3.1 Tumor-intrinsic signals and stromal scaffolds

Tumor-intrinsic signaling can be an early layer of niche initiation. Genetic drivers, epigenetic reprogramming, viral oncogenic programs, secretome output, metabolic adaptation, and stress-associated signaling can project tumor-cell states into the surrounding tissue. Oncogenic pathways can influence immune landscapes by altering PD-L1 expression, cytokine and chemokine output, CAF recruitment, myeloid accumulation, metabolic stress, and T-cell infiltration (45, 54–57). The point is not that each driver creates a separate niche, but that tumor-intrinsic programs bias the local ecological conditions under which niche assembly proceeds.

Stromal remodeling and ECM organization can convert these molecular biases into spatially durable tissue structures. CAFs, collagen deposition, fibronectin remodeling, matrix cross-linking, altered tissue mechanics, and matrix-associated signaling regulate immune-cell entry, retention, and exhaustion (23, 52, 58, 59). ECM is not merely a physical wall. It can influence cellular force transmission, molecular diffusion, chemokine distribution, vascular access, and the probability of direct contact between T cells and tumor cells. Representative spatial studies in breast, lung, and liver tumors show that CAF-myeloid-ECM interactions are associated with boundary structures that may restrict CD8+ T-cell entry, correlate with poor prognosis, or reduce response to anti-PD-1 therapy (60–63).

To make the evidence base explicit, this framework distinguishes representative primary spatial studies from broader mechanistic synthesis. For example, multiplexed ion beam imaging in triple-negative breast cancer revealed structured tumor-immune organization rather than random immune infiltration, while coordinated cellular-neighborhood analysis at the colorectal cancer invasive front linked local cellular neighborhoods to antitumoral immunity (21, 22). In lung tumors, spatial analysis of CAF matrix programs provided a concrete example of stromal positioning associated with T-cell exclusion (23). In hypoxic tumor regions, perturbation-oriented work showed that hypoxic niches can attract and sequester tumor-associated macrophages and cytotoxic T cells and reprogram them toward immunosuppression, moving the evidence beyond spatial proximity alone (44). These examples illustrate why this review separates primary spatial evidence, functional association, and perturbation-based validation rather than treating all niche claims as equally mature.

### 3.2 Chemokine-cytokine wiring and vascular-metabolic boundaries

Chemokine and cytokine gradients contribute to the spatial wiring of niche organization. They influence not only which immune cells enter tumors, but also where they accumulate, how long they remain, and with whom they interact (18, 51, 64). In immune-supportive contexts, chemokine-rich microregions can assemble dendritic cells, helper T cells, stem-like CD8+ T cells, and lymphoid structures into sites of adaptive immune coordination (12–14). In suppressive contexts, tumor- or stromal-derived cytokines and chemokines can recruit macrophages, Tregs, MDSCs, or neutrophils and stabilize chronic inflammatory circuits (65–68). The same class of signals can therefore produce either immune activation or immune suppression depending on cellular partners and tissue location.

Vascular dysfunction, hypoxia, and metabolic constraint can define the boundary conditions under which immune cells can function. Abnormal tumor vessels influence leukocyte trafficking, drug delivery, hypoxia, stromal remodeling, and suppressive-cell accumulation (53, 57, 69, 70). Hypoxia and HIF-associated programs reshape tumor cells, endothelial cells, fibroblasts, and myeloid cells, often reinforcing angiogenesis, metabolic adaptation, and immune suppression (71–73). Nutrient partitioning, glutamine utilization, lactate accumulation, acidity, and metabolic-epigenetic coupling can further weaken T-cell function, alter macrophage polarization, and modulate antigen visibility (74–77). These features are not merely background stressors; they may define local functional borders between immune-permissive and immune-restricted regions.

### 3.3 Myeloid-centered stabilization and tissue-resident imprinting

Myeloid-centered immunosuppressive programming may act as a central hub of niche stabilization. TAMs, monocytes, macrophage precursors, MDSCs, neutrophils, and related suppressive populations do not act as a static cell mass. They can be continuously programmed by tumor-derived signals, bone marrow myelopoiesis, stromal cues, metabolic states, and reciprocal interactions with exhausted T cells and regulatory lymphocytes (2, 78, 79). Recent work has moved beyond the simple M1/M2 dichotomy, showing that myeloid cells differ by origin, spatial position, temporal state, and metabolic program. Their importance lies in their potential capacity to connect angiogenesis, ECM remodeling, antigen-presentation failure, T-cell exhaustion, metastasis, and therapeutic resistance (80–82).

Tissue-resident immune programs and local immune imprinting help explain why these general mechanisms do not yield identical niches across organs. Resident macrophages, microglia, alveolar macrophages, liver macrophages, tissue-resident memory T cells, local stromal populations, and organ-specific barrier systems can provide pre-existing immune architectures into which tumors emerge (83–86). These resident programs may support immune surveillance, as when trained alveolar macrophage states enhance antitumor responses, or they may be repurposed into protumor niches, as when resident macrophages, tumor-derived signals, and suppressive recruitment programs cooperate during local or premetastatic niche formation (87–89). Organ specificity should therefore be treated not as an added variable after common TME mechanisms, but as a constitutive condition that shapes niche identity from the beginning.

## 4 Organ-specific niche architectures: brain and lung as two paradigms

Brain and lung tumors are used here as a useful contrast for an organ-specific niche framework (24–29). Brain tumors are presented here as an immune-restricted niche paradigm, shaped by central nervous system borders, resident microglia, glial-immune communication, and perivascular, hypoxic, and necrotic microdomains (24–26, 44, 90, 91). Lung tumors are presented here as an inflammation-primed niche paradigm, shaped by continuous environmental exposure, epithelial injury-repair cycles, chronic pulmonary inflammation, TLS-associated lymphoid organization, and therapy-remodeled immune compartments (27–33). These paradigms should not be understood as mutually exclusive categories for all tumors, but as two ends of a conceptual spectrum: one organized by immune restriction in a relatively protected tissue, the other by chronic exposure and inflammatory conditioning.

**Figure 2:**
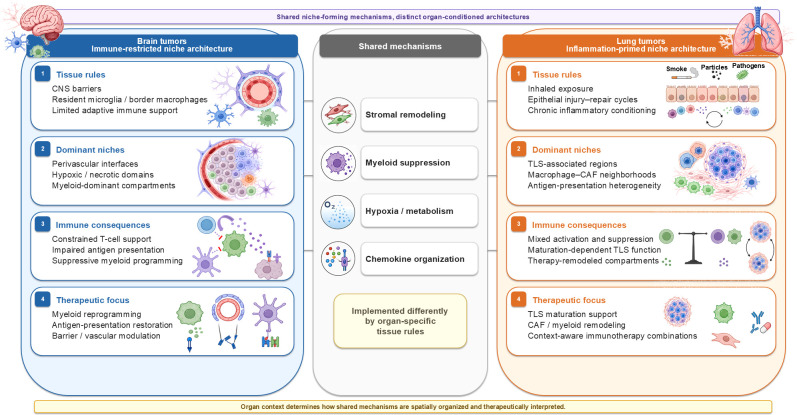
Brain and lung tumors as two paradigms of organ-specific immunological niche architecture. The schematic provides a simplified side-by-side comparison of immune-restricted brain tumor niches and inflammation-primed lung tumor niches. Brain tumors are summarized in terms of CNS barriers, resident microglia and border macrophages, perivascular, hypoxic, and necrotic domains, constrained T-cell support, impaired antigen presentation, suppressive myeloid programming, and selected therapeutic focuses. Lung tumors are summarized in terms of inhaled exposure, epithelial injury-repair cycles, chronic inflammatory conditioning, TLS-associated regions, macrophage–CAF neighborhoods, antigen-presentation heterogeneity, mixed immune activation and suppression, maturation-dependent TLS function, therapy-remodeled compartments, and selected therapeutic focuses. The schematic emphasizes that shared niche-forming mechanisms are implemented differently by organ-specific tissue rules. This figure is an author-prepared conceptual schematic, not an AI-generated image, and does not represent quantitative spatial data. CNS, central nervous system; TLS, tertiary lymphoid structure; CAF, cancer-associated fibroblast.

**Figure 2** presents a side-by-side conceptual comparison of brain and lung tumor immunological niche architectures. It illustrates how shared niche-forming mechanisms, including stromal remodeling, myeloid suppression, hypoxia, metabolic stress, chemokine organization, antigen-presentation state, and vascular access, are implemented differently by CNS immune restriction and pulmonary inflammatory conditioning. This figure is an author-prepared conceptual schematic and does not represent quantitative spatial data.

### 4.1 Brain tumors as immune-restricted niche architecture

In brain tumors, immune niches are embedded within specialized central nervous system tissue borders and resident myeloid programs. Glioblastoma is not adequately described as simply “cold.” It is spatially compartmentalized and myeloid-dominated, with microglia, CNS-border macrophages, monocyte-derived macrophages, perivascular interfaces, and tumor-instructed suppressive programs forming the key cellular architecture (24, 25, 91, 92). Multiomic spatial studies show that brain parenchyma, meninges, choroid plexus, and perivascular spaces contain layered myeloid distributions before tumor development, indicating that immune restriction in brain tumors is superimposed on pre-existing CNS immune geography (25).

A concrete example is provided by single-cell spatial immune mapping of primary and metastatic brain tumors, which showed that brain tumor immunity is organized into spatially distinct immune landscapes rather than a uniform cold compartment (38). This type of evidence supports the niche framework at the level of primary spatial evidence because it identifies local immune neighborhoods and myeloid-dominated patterns, but it should not by itself be interpreted as proof that a specific niche organizer is causally responsible for therapeutic resistance. The CNS-border multiomic study further strengthens the organ-conditioned interpretation by showing that layered myeloid distributions exist across meninges, choroid plexus, parenchyma, and perivascular spaces before tumor-specific remodeling occurs (25).

Perivascular, hypoxic, and necrotic microdomains give this restriction a concrete spatial form. Perivascular niches can integrate endothelial cells, pericytes, stromal-like cells, macrophages, tumor stem-like states, and therapy-resistant subclones (90, 94, 95). With progression, abnormal perfusion and hypoxia are associated with myeloid migration, TAM sequestration, chemokine secretion, and immunosuppressive polarization (44, 96, 97). Perinecrotic regions further stabilize monocyte-derived macrophage states, vascular hyperpermeability, drug-access limitations, efferocytosis, and invasion-related inflammatory programs (91, 98–100). Brain tumor niches may therefore restrict immunity through coordinated spatial barriers: limited T-cell support, myeloid dominance, vascular-immune coupling, metabolic stress, and necrosis-associated suppression.

These features help explain why checkpoint blockade alone has limited efficacy in glioblastoma. The problem is not only insufficient immune infiltration or low antigenicity. T cells that enter the lesion frequently encounter niches that fail to sustain activation, while myeloid cells can be epigenetically, metabolically, or hypoxia-programmed toward suppressive states (26, 101–103). Experimental strategies that reprogram glioma-associated myeloid cells, enhance antigen presentation, activate cGAS-STING-type I interferon signaling, or disrupt neuron-tumor-immune coupling support the possibility that successful immunotherapy may require coordinated remodeling of myeloid, vascular, antigen-presenting, and T-cell-supportive compartments rather than isolated checkpoint release (104–106).

### 4.2 Lung tumors as inflammation-primed niche architecture

Lung tumors, by contrast, arise in a tissue constantly exposed to inhaled particles, pollutants, microbial products, smoking-related toxicants, infection, and repeated epithelial injury. The lung niche may therefore be shaped by pre-existing immune conditioning before overt tumor expansion. AT2 cells, airway and alveolar epithelial cells, resident myeloid populations, and lymphoid populations participate in recurrent injury detection and repair; when these programs persist, restorative inflammation may shift toward unresolved remodeling, barrier dysfunction, and tumor-permissive immune organization (27, 28, 107, 108). This supports interpreting pulmonary tumor niches in the context of prolonged exposure and failed repair, rather than solely as consequences of malignant-cell outgrowth.

Spatial organization in lung cancer often becomes visible through lymphoid and myeloid compartments. Spatial multi-omics of lung adenocarcinoma precursors and single-cell mapping of tumor development show that immune organization diversifies from premalignant stages through invasive disease, with Tfh-like cells, B-cell programs, dysfunctional CD8+ T cells, macrophage-CAF neighborhoods, and TLS-associated structures occupying distinct tissue positions (30–33). TLSs are particularly important in lung cancer, but their meaning depends on maturation, germinal-center status, anatomical context, and neighboring stromal or myeloid states (8–11). Mature TLSs may support antigen presentation and adaptive immunity, whereas immature or spatially constrained lymphoid aggregates may not produce equivalent benefit.

More specifically, spatial and multiomic analysis of human and mouse lung adenocarcinoma precursors showed that immune organization is already remodeled during premalignant-to-invasive progression and identified TIM-3 as a potential interception target, illustrating how a candidate niche can emerge before advanced disease (30). Single-cell mapping of lung adenocarcinoma development further connected Tfh-dependent TLS programs with antitumor immunity, while studies of TLS maturation showed that germinal-center status and draining-lymph-node context influence the functional meaning of intratumoral lymphoid structures (31, 32). Neoadjuvant chemoimmunotherapy studies in NSCLC also show that treatment can remodel macrophage, fibroblast, T-cell, and antigen-presentation compartments, supporting the view that lung tumor niches are dynamically therapy-conditioned rather than static baseline features (33).

Chronic pulmonary diseases and inflammatory precursors may further shape niche architecture. Smoking, COPD, idiopathic pulmonary fibrosis, and other long-term inflammatory conditions can leave epithelial, stromal, vascular, and immune imprints that resemble preassembled niche conditions (29, 34–36). In such settings, tumor immunity should be interpreted in relation to the tissue history in which malignant transformation occurs. This inflammation-primed logic also affects therapeutic response. Lung tumors may contain immune-reactive structures that support checkpoint blockade, but they may also develop antigen-presentation defects, exhausted T-cell states, macrophage-CAF barriers, and therapy-induced suppressive remodeling (33, 63, 109–112).

### 4.3 What the brain-lung comparison contributes

The brain–lung comparison shows that organ specificity determines how shared niche-forming mechanisms are implemented (24–36). In the brain, CNS borders, vascular interfaces, and glial–myeloid programs constrain sustained local immune activation (24–26, 44, 90, 91); in the lung, environmental exposure and recurrent injury–repair cycles may generate both immune-permissive lymphoid structures and suppressive inflammatory–stromal neighborhoods (27–36, 63, 111). Consequently, interventions targeting myeloid cells, hypoxia, the ECM, or immune checkpoints may have different effects across organs because tissue barriers, resident immune programs, and inflammatory history differ (33, 63, 102–106, 110, 112).

**Table 4** summarizes these contrasts without reducing brain tumors to uniformly immune-cold lesions or lung tumors to uniformly TLS-rich or checkpoint-responsive lesions. Both organs contain mixed immune-permissive and immune-restrictive states; the key distinction lies in the spatial organization and evidence-supported function of local immune programs rather than in immune-cell abundance alone (3, 19, 24–36, 44, 63, 91, 112).

## 5 Comparative extensions beyond brain and lung

Brain and lung tumors provide the main paradigms, but other solid tumors illustrate how additional tissue rules may modify shared niche mechanisms (113–124). Oral, liver, and pancreatic tumors are useful comparative cases because they represent three different niche-forming logics: mucosal-microbial remodeling, tolerogenic-metabolic conditioning, and desmoplastic immune exclusion (113–124). These cases are used as focused comparators rather than exhaustive disease-specific reviews.

Oral squamous cell carcinoma, a subtype of head and neck squamous cell carcinoma, arises at oral mucosal barrier surfaces, where epithelial injury, oral and intratumoral microbiota, dietary and chemical exposures, mechanical irritation, and recurrent inflammation converge (113–116, 125–127). A niche-centered interpretation should avoid a simplistic single-microbe causality model (114, 125, 126). A more cautious interpretation is that microbial communities, epithelial damage, innate signaling, macrophage polarization, CAF activation, and T-cell dysfunction may participate in reciprocal remodeling loops (113–115, 125, 126). Oral tumor immunity is therefore conditioned by whether barrier-surface inflammation supports immune recognition or is converted into durable suppressive neighborhoods. Spatial studies of OSCC and broader HNSCC cohorts show that malignant epithelial cells, fibroblasts, myeloid populations, MDSCs, and T cells form structured local interactions rather than homogeneous immune infiltrates (115, 116, 127).

Liver tumors arise in a physiologically tolerogenic and metabolically active organ. HCC niches are shaped by portal circulation, viral persistence, steatotic liver disease, cirrhosis, gut-liver signaling, fibrosis, metabolic flux, and endothelial-macrophage-stellate cell interactions (117, 118, 128, 129). This context helps explain why hepatic immune escape may emerge from mechanisms that normally prevent excessive inflammatory damage. Fibrosis, stellate-cell activation, endothelial programs, Tregs, macrophages, bile acid metabolism, and metabolic stress may together contribute to niches that limit cytotoxic immunity and modify response to immunotherapy (118–120, 128–130). The liver case therefore illustrates how immune tolerance and metabolic filtering can be repurposed into tumor-supportive architecture.

Pancreatic ductal adenocarcinoma exemplifies desmoplastic immune exclusion. Its niche is organized through dense ECM, CAF heterogeneity, macrophage and neutrophil circuits, hypoxia, altered mechanics, metabolic stress, and restricted T-cell penetration (121–123, 131). PDAC is not resistant to immunotherapy merely because it lacks immune targets. Its immune failure is embedded in stromal and physical conditions that determine whether effector cells can enter, persist, and function (124, 132). This supports using pancreatic cancer as a critical comparator for understanding why niche-directed therapy may require stromal, myeloid, metabolic, and delivery-based strategies before checkpoint release can become effective.

Across these tumors, shared mechanisms recur: stromal remodeling, myeloid suppression, chemokine-guided positioning, metabolic constraint, TLS variability, microbial or inflammatory conditioning, and therapy-induced remodeling. What differs is the tissue-specific implementation. Oral tumors are shaped primarily by barrier injury and microbial-immune crosstalk (113–116, 125–127); liver tumors by immune tolerance, fibrosis, and metabolic filtering (117–120, 128–130); and pancreatic tumors by desmoplastic exclusion and fibroblast-myeloid restriction (121–124, 131, 132). Broader pan-cancer and cross-tumor studies further support the view that myeloid states, stromal remodeling, TLS organization, and immune compartments vary substantially across tumor types (133–136). The comparative lesson is that immune niches are neither fully unique nor fully interchangeable. Shared components become distinct niches only when organized by local tissue rules.

## 6 Inflammatory parallels: chronic tissue programs as comparative contexts for tumor immunological niches

Inflammatory parallels are retained in this review as a comparative and hypothesis-generating tool, not as direct proof that chronic inflammatory diseases and tumors are biologically identical. To avoid conceptual overextension, this section separates three evidentiary categories: direct evidence generated in tumors, analogical evidence derived from chronic inflammation or fibrosis, and hypotheses that require tumor-specific spatial and functional validation.

### 6.1 Direct tumor evidence and organ-conditioned interpretation

Direct tumor evidence in brain tumors includes single-cell spatial mapping of primary and metastatic lesions, which identifies spatially distinct immune landscapes and myeloid-dominated neighborhoods (38). These tumor-specific findings can be interpreted in the context of multiomic analyses of CNS borders, which reveal layered myeloid distributions across the meninges, choroid plexus, parenchyma, and perivascular spaces before tumor-specific remodeling occurs (25). More broadly, CNS tissue-border organization, resident myeloid programming, vascular interfaces, injury-associated signals, and local metabolic conditions provide an organ-conditioned context for interpreting brain tumor immune architecture (24–26, 44, 83, 90, 91, 106). Such parallels do not prove that neuroinflammatory or neurodegenerative disease mechanisms directly drive glioma immunity; rather, they help explain why antitumor immunity may fail even when inflammatory or innate immune activity is detectable (24, 26, 44, 91, 102, 103). In this review, spatial or functional findings from tumor samples and tumor models are treated as direct tumor evidence, whereas observations from neuroinflammation, COPD, pulmonary fibrosis, wound repair, or mucosal inflammatory disease are treated as analogies unless the same circuit has been validated within a tumor-specific spatial or functional context.

### 6.2 Analogical evidence from chronic inflammation and fibrosis

Lung tumors illustrate a second inflammatory parallel through chronic pulmonary injury and failed repair (27–36, 107, 108). The lung is continuously exposed to inhaled particles, pollutants, pathogens, and smoking-related toxicants, and repeated epithelial injury can activate AT2-cell repair programs, macrophage remodeling, fibroblast activation, vascular changes, and lymphoid aggregate formation (27, 28, 34, 35, 107, 108). COPD, pulmonary fibrosis, and other chronic inflammatory lung diseases may therefore create tissue histories that resemble niche-relevant tissue conditions (29, 34–36). When malignant transformation occurs within such a context, tumor-associated immune niches may reflect both immune-activating and immune-suppressive features: TLSs and antigen-presenting compartments may support local immunity, whereas macrophage-CAF neighborhoods, antigen-presentation heterogeneity, and unresolved inflammatory repair may constrain durable therapeutic response (30–33, 63, 110–112).

### 6.3 Hypotheses requiring tumor-specific validation

Fibrosis and wound-healing programs offer a broader cross-organ parallel (121, 128, 137). In physiological repair, fibroblast activation, ECM deposition, angiogenesis, macrophage recruitment, inflammatory signaling, and TGF-β signaling are temporally regulated to restore tissue integrity (128, 137). In tumors, similar features may become spatially enriched and immunologically maladaptive (23, 121, 128, 131, 137). CAF-rich boundaries, collagen remodeling, suppressive macrophage-neutrophil circuits, hypoxia, and metabolic stress are consistent with a model in which repair-like programs contribute to durable immune-exclusion niches (23, 44, 121, 128, 131, 137, 138).

This analogy is useful only when its limits are clearly stated. Inflammatory parallels should guide hypothesis generation, mechanistic interpretation, and prioritization of validation experiments, but they should not be treated as direct evidence for tumor niche causality unless supported by tumor-specific spatial mapping, functional readouts, perturbation experiments, or clinical biomarker associations (3, 19, 23, 44). This qualification is particularly important when translating repair-like or fibrosis-like programs into niche-directed therapeutic claims.

## 7 Functional consequences and therapy resistance of tumor immunological niches

### 7.1 Spatial immune exclusion and T-cell dysfunction

A central reason to study immunological niches is that they may help explain how antitumor immunity fails, persists in a dysfunctional state, or can be restored. Four functional consequences recur across solid tumors: spatial immune exclusion, antigen-presentation failure, myeloid-stromal suppressive feedback, and niche-mediated therapy resistance. These consequences are not separate endpoints; they often reinforce one another. A stromal barrier may simultaneously prevent T-cell contact, recruit suppressive myeloid cells, impair dendritic-cell function, stabilize hypoxia, and reduce response to checkpoint blockade (23, 137–140).

Spatial immune exclusion is a niche-level state rather than a simple absence of T cells. Immune-excluded tumors may contain detectable lymphocytes, but these cells are retained in stromal regions, tumor margins, perivascular zones, or other non-contact compartments. CAF subsets, ECM alignment, collagen stiffness, myeloid-neutrophil circuits, metabolic stress, and organ-specific myeloid programs can all reduce productive contact between T cells and tumor cells (23, 44, 59, 111, 137–139). This means that reinvigorating exhausted T cells may be insufficient if the local tissue architecture still prevents their access or function.

The CAF-myeloid barrier provides a useful primary-study example of this principle. In human lung tumors, spatial positioning and matrix programs of CAFs were linked to T-cell exclusion, showing that the relevant niche feature is not merely CAF abundance but the local relationship among fibroblast state, matrix architecture, and CD8+ T-cell access (23). This example supports an interpretation beyond simple colocalization because the spatial arrangement is connected to a defined immune consequence, although therapeutic actionability still requires context-specific validation.

### 7.2 Antigen-presentation failure and immune ignorance

Antigen-presentation failure can create immune-ignorant niches in which tumor antigens are present but are not effectively displayed, sensed, or converted into productive T-cell priming. This can occur through tumor-cell-intrinsic repression of MHC-related programs, antigen-processing defects, impaired dendritic-cell maturation or migration, altered interferon or innate immune sensing pathways, microbiota-associated modulation of immune priming, or local metabolic and stromal constraints affecting antigen-presenting function (12, 105, 112, 141–144). Importantly, antigen-presentation failure is not uniform across organs; different tissue contexts may generate immune ignorance through distinct tumor-cell-intrinsic, myeloid, stromal, inflammatory, or metabolic routes (24–26, 30, 33, 66, 102, 121–124, 139, 141–143).

A complementary example comes from intratumoral dendritic cell-CD4+ T-cell niches in hepatocellular carcinoma, where single-cell and spatially informed immune profiling linked local DC-helper T-cell organization to CD8+ T-cell differentiation following PD-1 blockade (12). This finding illustrates why antigen presentation should be treated as a niche-level process: the relevant question is not only whether antigen-presenting cells are present, but whether they are positioned with helper and effector lymphocytes in a configuration that may help license productive antitumor immunity. Similar logic applies to tumors with IFN/MHC defects or therapy-induced antigen-presentation remodeling, where immune ignorance may persist despite immune-cell infiltration.

### 7.3 Myeloid-stromal feedback and niche-mediated resistance

Myeloid-stromal microdomains may stabilize suppression through reciprocal feedback. CAFs can recruit and polarize macrophages or neutrophils; myeloid cells can reinforce ECM remodeling and T-cell dysfunction; tumor-derived metabolites, cytokines, inflammatory signals, adenosine pathways, and checkpoint-related ligands can maintain these interactions (23, 62, 63, 67, 68, 137, 139, 140, 145). These circuits also provide a direct route through which repair-like or inflammation-associated tissue programs become locked into persistent suppressive architectures.

Niche-mediated resistance may be understood as a phenotype of local immune ecology when spatial evidence is linked to functional or treatment-response evidence. During immunotherapy, chemotherapy, radiotherapy, targeted therapy, or anti-angiogenic treatment, tumors may lose antigen visibility, reinforce stromal exclusion, select resistant tumor-cell states, remodel vasculature, intensify myeloid suppression, or generate therapy-induced inflammatory feedback (33, 57, 146–150). This interpretation extends the cancer immunoediting framework from tumor-cell immunogenicity to spatially organized immune environments. Treatment failure is rarely explained by one immune checkpoint alone; it often reflects coordinated changes in immune recognition, tissue access, suppressive feedback, and local metabolic constraint.

## 8 Therapeutic remodeling and reprogramming of tumor immunological niches

### 8.1 From immune activation to niche conversion

Therapeutic reprogramming aims to convert suppressive, excluded, or metabolically constrained niches into configurations more permissive to antitumor immunity. Potential approaches include antigen-presentation restoration, myeloid reprogramming, dendritic-cell activation, regulatory T-cell modulation, TLS maturation, vascular normalization, metabolic intervention, stromal state switching, local delivery, nucleic-acid-based therapies, and spatially guided combination strategies (12, 15, 76, 78, 98, 104, 105, 112, 151–153). The objective is not nonspecific immune intensification, but evidence-supported modification of the dominant local barrier affecting immune access, antigen recognition, suppressive feedback, and effector persistence (3, 6, 12, 19, 23, 44, 135). Accordingly, niche remodeling should be assessed through spatial pharmacodynamic changes as well as conventional response metrics (19, 20, 33, 40, 57, 110, 150).

### 8.2 Three therapeutic logics: release, access, and licensing

Niche-directed therapy can be organized into three complementary therapeutic logics: release, access, and licensing (6, 143). Release strategies aim to remove inhibitory signals or suppressive cellular programs, including immune-checkpoint blockade, regulatory T-cell modulation, myeloid reprogramming, and inhibition of suppressive cytokine or metabolite circuits (57, 78, 82, 102–104, 119, 123, 151). Access strategies aim to improve the physical, stromal, metabolic, and vascular conditions required for immune-cell entry and tumor-cell contact, including stromal remodeling, ECM targeting, CAF/TGF-β targeting, vascular normalization, hypoxia correction, local delivery, and modulation of fibroblast-myeloid barriers (23, 69, 90, 98, 131, 132, 137, 152, 153). Licensing strategies aim to restore the immune-recognition machinery that allows antitumor responses to begin and persist, including dendritic-cell activation, antigen-presentation restoration, IFN/MHC pathway recovery, STING/type I IFN activation, and TLS maturation (9, 12, 105, 112, 135, 141, 142).

These strategies are not equally mature clinically. **Table 5** therefore separates established clinical evidence, clinical biomarker evidence, early clinical rationale, and preclinical or hypothesis-generating rationale before linking each strategy to organ-specific niche barriers.

### 8.3 Organ-tailored combination strategies

Organ-tailored strategies follow from this logic. In brain tumors, niche reprogramming may require coordinated targeting of myeloid suppression, vascular-immune barriers, antigen-presentation states, and neuron-glial-immune circuits (24–26, 44, 90, 98, 102–106). In lung tumors, strategies must account for TLS maturation, chronic inflammatory conditioning, antigen-presentation heterogeneity, and therapy-induced macrophage-CAF remodeling (27–36, 63, 110, 112). In oral tumors, microbial-inflammatory-myeloid interactions may influence response (113–116, 125–127, 154). In liver tumors, fibrosis, tolerance, vascular organization, and metabolism may define dominant constraints (117–120, 128–130, 139). In pancreatic tumors, desmoplasia, CAF-myeloid loops, hypoxia, and drug-access barriers remain central (15, 121–124, 131, 132). Spatial biomarkers, organoid models, single-cell and spatial profiling, computational pathology, and functional validation can be integrated to identify which niche state is actionable rather than merely descriptive (19, 20, 40, 43, 145, 155).

Accordingly, niche-directed therapeutic actionability should be treated as evidence-dependent. Checkpoint blockade has established clinical relevance in selected tumor types, particularly some lung cancer settings, whereas myeloid reprogramming, STING/type I IFN activation, antigen-presentation restoration, TLS induction, CAF/TGF-β targeting, metabolic modulation, and vascular normalization remain variably supported across organs. Niche-directed therapy should therefore be presented as a graded translational framework rather than a uniformly mature clinical approach.

## 9 Limitations and future perspectives

Several limitations should be acknowledged. First, spatial association does not prove causal niche function. Proximity between cell types, or enrichment of a spatial signature, must be linked to recurrent interaction and an evidence-supported functional consequence—through functional experiments, longitudinal sampling, or treatment-response data—before a candidate niche is interpreted as functionally validated (3, 19, 40). Second, many spatial datasets remain limited by sampling bias, platform differences, fixation and imaging constraints, incomplete protein or metabolic readouts, and small clinical cohorts (19, 40, 101). Third, organ-specific interpretation can be weakened when evidence from inflammatory disease, tissue repair, or fibrosis is imported without clearly defining whether it represents direct tumor evidence, analogy, or hypothesis generation. Such evidence is useful for mechanistic interpretation, but it should not be treated as direct proof of tumor niche behavior without tumor-specific validation (27–29, 34–36, 128, 137). Fourth, therapeutic actionability should be reserved for cases in which a niche state is targetable, monitorable, and linked to treatment outcome or pharmacodynamic change; otherwise, such claims should be framed as candidate vulnerabilities or preclinical rationales.

Future studies should therefore move from descriptive niche mapping to functional niche validation. A practical framework would involve four steps: defining the dominant niche architecture through spatial and single-cell profiling; testing whether that niche causally drives immune suppression, metastatic progression, or therapy resistance; selecting an intervention that targets the dominant barrier, such as myeloid modulation, stromal remodeling, hypoxia correction, metabolic rewiring, antigen-presentation restoration, engineered delivery, or organ-tailored immunotherapy; and monitoring spatial pharmacodynamic changes during treatment rather than relying only on bulk endpoint measures such as tumor shrinkage (3, 15, 19, 23, 33, 40, 43, 101, 110, 112, 139, 150, 153). Through this approach, tumor immunological niches may progress from descriptive labels of spatial complexity to functionally validated and potentially actionable units for precision immunotherapy.

A further challenge is to move from static niche classification to spatiotemporal niche analysis. Within the same tumor, perivascular, hypoxic, myeloid–stromal, antigen-presentation, and TLS-associated niches should not be interpreted as isolated compartments. Rather, they may coexist, overlap, interact, and undergo coordinated or compensatory remodeling during tumor progression and under therapeutic pressure (17, 19, 33, 44, 78, 112, 149). For example, therapy may reduce one suppressive compartment while promoting compensatory myeloid, stromal, antigen-presentation, or metabolic remodeling elsewhere in the tumor (33, 57, 149, 150). Longitudinal sampling, spatially resolved pharmacodynamic profiling, and paired pre- and post-treatment analyses are therefore needed to determine how the relative dominance and functional states of different niches shift over time (19, 33, 40, 149). This spatiotemporal view may help distinguish stable niche architectures from transient treatment-induced states and guide the selection of organ-tailored combination strategies.

## 10 Conclusion

Tumor immunological niches may refine the TME framework by connecting spatial organization, organ specificity, inflammatory parallels, evidence strength, and therapeutic vulnerability. The value of this framework lies not in proposing another general taxonomy, but in explaining why similar immune, stromal, vascular, and molecular components can produce different outcomes under distinct tissue rules. A niche-centered view therefore shifts attention from which immune cells are present to how local programs are organized and functionally stabilized, and to how strongly their inferred roles are supported by evidence. Brain and lung tumors illustrate immune-restricted and inflammation-primed architectures, whereas oral, liver, and pancreatic tumors show how additional tissue rules reshape shared mechanisms. Future progress will depend on functional and longitudinal validation of spatiotemporal niche architectures to distinguish stable organ-conditioned niches from transient therapy-induced states, as well as on translating validated niche states into evidence-graded biomarkers and organ-tailored therapeutic strategies.
